# The brain-lung axis: Bridging neurological and respiratory disorders via neural-immune-microbial dialogue

**DOI:** 10.1016/j.cjtee.2025.10.006

**Published:** 2026-02-05

**Authors:** Wenpei Yang, Xiaoyu Shi, Lizheng Xie, Hao Zhang, Lina Shen, Li Pan, Shi Feng, Xiang Mao, Xiao Wu

**Affiliations:** a Department of Neurosurgery, The First Affiliated Hospital of Anhui Medical University, Hefei, 230022, China; b Innovation and Entrepreneurship Laboratory for College Students, Anhui Medical University, Hefei, 230022, China; c Department of General Practice, Peking University Third Hospital, Beijing, 100191, China; d Department of Emergency, The First Affiliated Hospital of Anhui Medical University, Hefei, 230022, China

**Keywords:** Brain-lung axis, Neural pathways, Neuroinflammation, Pathophysiology, Treatment, Secondary disorders, Complications

## Abstract

The human brain maintains intricate interconnections with various peripheral organs. Recent scientific inquiry has substantiated the existence of the gut-brain axis; nevertheless, emerging evidence suggests that the brain and lungs engage in bidirectional communication through multiple pathways, thereby giving rise to the conceptualization of a brain-lung axis. Studies indicate the presence of crosstalk between the central nervous system and the lungs, mediated by the lung microbiome, neural pathways, metabolite signaling, and immune pathways. This bidirectional communication between the brain and lungs is further implicated in the pathogenesis of several diseases: traumatic brain injury, stroke, and other cerebral disorders can precipitate pulmonary injury; conversely, severe pulmonary conditions, such as acute respiratory distress syndrome and chronic obstructive pulmonary disease, can exacerbate neuroinflammation, intensify brain damage, impair neurological function, and contribute to adverse prognoses. Exploring the brain-lung axis not only facilitates a multifaceted understanding of disease progression, but also unveils critical targets for therapeutic intervention. Research into the brain-lung axis provides novel perspectives for deciphering underlying pathological mechanisms, developing diagnostic methodologies, and formulating treatment strategies. It further establishes a theoretical foundation for cross-organ targeted therapies, holding promise for ameliorating patient outcomes and promoting the advancement of integrated diagnostic and therapeutic approaches for respiratory and neurological disorders.

## 1 Introduction

**Fig. 1:**
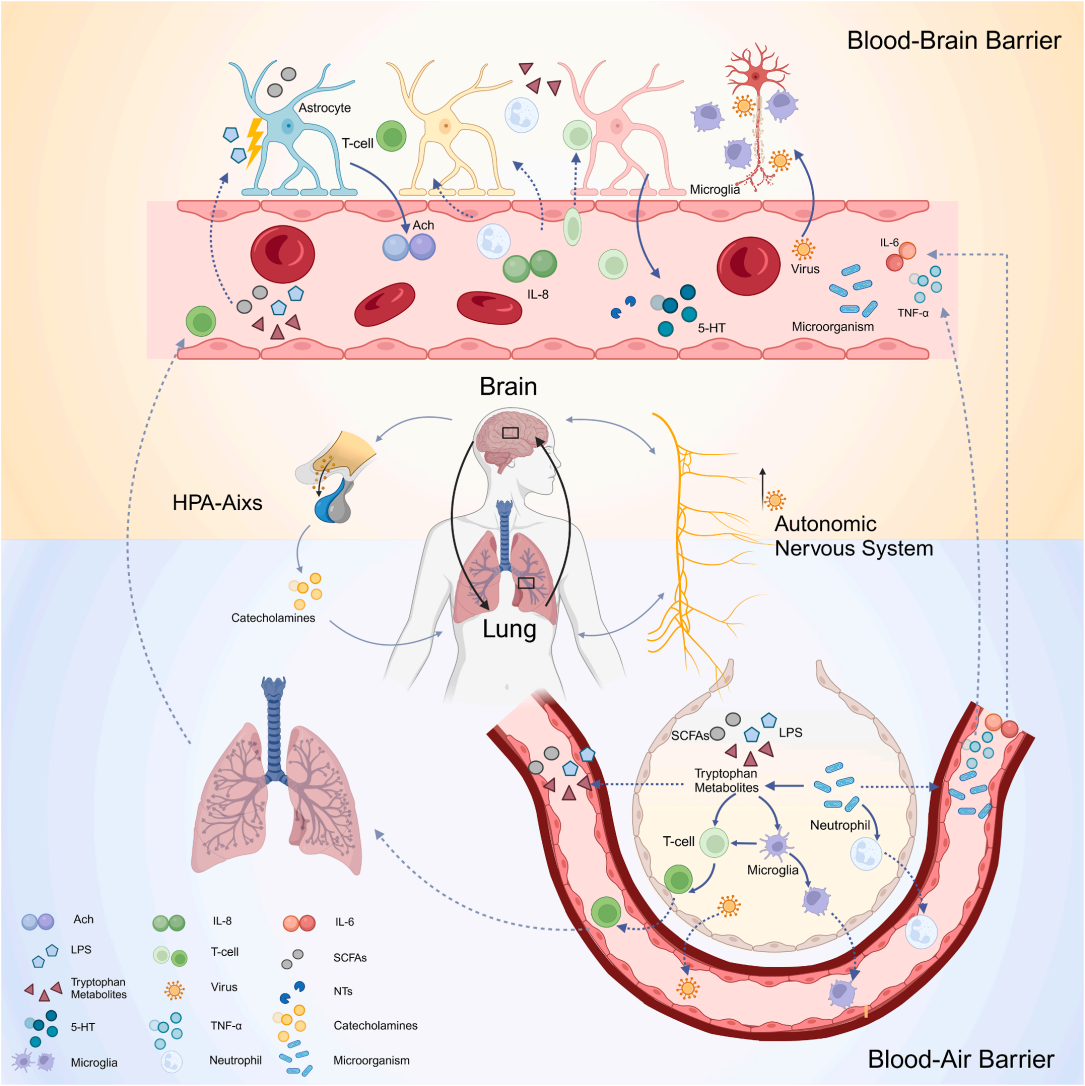
Schematic diagram of the brain-lung axis interaction network. The communication between the brain and lungs is mediated through direct microbial translocation and metabolite signaling, as well as bidirectional regulation of the autonomic nervous system. HPA: hypothalamic-pituitary-adrenal axis; IL: interleukin; NTs: neurotransmitters; TNF-α: tumor necrosis factor-αpha; Ach: acetylcholine; LPS: lipopolysaccharide; SCFAs: short-chain fatty acids; 5-HT: 5-hydroxytryptamine. Created with BioRender.com.

The brain-lung axis constitutes a bidirectional pathway facilitating dynamic interplay between the central nervous system (CNS) and the respiratory system, whose dysregulation is intricately associated with the pathological progression of numerous diseases. Dysbiosis of the lung microbiome influences the susceptibility to autoimmune inflammation of the brain, participates in CNS autoimmune processes, and is implicated in conditions such as Alzheimer's disease (AD) and multiple sclerosis (MS). Within neural pathways, the autonomic nervous system exerts bidirectional modulatory effects in asthma, neurogenic pulmonary edema (NPE), and chronic obstructive pulmonary disease (COPD) by regulating airway reactivity, propagating inflammatory signals, and mediating neurotransmitter secretion. Regarding metabolite pathways, neuropeptides and extracellular vesicles exacerbate pathological damage in acute respiratory distress syndrome (ARDS) and pneumonia by mediating pulmonary vascular permeability, facilitating the dissemination of inflammatory cytokines, and promoting alveolar microthrombosis. The immune pathway, predominantly orchestrated by microglia, features activation states modulated by the lung microbiome; these activated microglia interact with peripheral immune cells via the release of pro-inflammatory mediators, collectively driving neuroinflammation and the systemic inflammatory response characteristic of brain-lung axis-related disorders (Fig. 1).

## 2 Search strategy

The articles used in this review were retrieved through an electronic search of the PubMed database for publications from 2015 to 2025. The search was performed using the following key terms and Boolean operators: (((brain) AND (lung)) OR (TBI)) OR (stroke). To achieve a comprehensive search, we did not restrict the search terms to specific disease mechanisms or study designs. We expanded the search range by incorporating synonymous or related keywords, such as "traumatic brain injury" for TBI and "cerebral infarction" for stroke, to enhance sensitivity. The initial results were further screened based on the title and abstract, focusing on those containing immune response or inflammation-related information.

## 3 Composition of the brain-lung axis

### 3.1 Lung microbiome

Recent investigations have revealed that although the lung microbiome possesses comparatively low biomass, with an abundance of approximately 10^8^ CFU/g, in contrast to the gut microbiota's 10^13^ – 10^14^ CFU/g, even relatively minor external perturbations may suffice to induce its dysbiosis. Owing to the lung's unique integration within the circulatory system and the distinctive architecture of the blood-air barrier, the distance microbial signaling molecules must traverse to reach the bloodstream is considerably shorter in the lung than in the intestine. This distance is minimized particularly in the alveolar region to optimize gas exchange: the boundary between alveolar epithelial cells and the capillary endothelial layer consists solely of a shared basement membrane.^1^ In the context of pulmonary disease, alterations occur in the composition, abundance, and functional capacity of the microbiota. Notably, studies have demonstrated that shifts in the lung microbiome influence cerebral susceptibility to autoimmune inflammation.

Research in microbial endocrinology demonstrates that the lung microbiome or their metabolic products may translocate directly across the alveolar-capillary barrier. Upon entering the systemic circulation, these components can reach the CNS, where they may exert functional effects. Utilizing an experimental model of Sprague-Dawley rats subjected to controlled cortical impact to induce cortical and hippocampal damage, investigators observed that severe TBI induces significant structural alterations in the lung microbiome. This includes the translocation of gut microbiota to the lungs, which subsequently precipitates secondary pulmonary infections following severe TBI.^2^ In a study by Ma et al.^3^ employing a rat model of experimental autoimmune encephalomyelitis with pulmonary involvement, neomycin administration was found to alter the lung microbiome, compromise the integrity of both the blood-brain barrier (BBB) and the pulmonary endothelial barrier—as evidenced by reduced expression of the associated proteins plasmalemma vesicle associated protein 1 and zona occludens 1—and facilitate bacterial translocation from the lungs into the bloodstream, thereby impacting brain function. The modulation of the CNS by the lung microbiota via the lung-brain axis occurs primarily through the pulmonary brain-derived neurotrophic factor (BDNF)-TrkB signaling pathway. Emerging evidence indicates that inhaled administration of a TrkB agonist can activate pulmonary vagal afferents, demonstrating therapeutic potential for migraine treatment.^4^

Dysbiosis of the lung microbiome induced by environmental alterations may contribute to TBI and subsequent behavioral deficits. Lu et al.^5^ observed that infection of aged AD mice with Gram-negative respiratory pathogens resulted in pronounced glial cell activation and β-amyloid deposition. Exposure to infection accelerated AD pathological alterations and cognitive impairment. Furthermore, respiratory viral or bacterial infections can induce pulmonary dysbiosis in MS patients, thereby influencing inflammatory regulation in MS. In murine models, airway inoculation with *Pseudomonas aeruginosa*, a pathogen known to induce pneumonia, also elicits anxiety-like behaviors by disrupting the BBB in mice. This finding is further supported by clinical reports demonstrating that *Pseudomonas aeruginosa* infection induced by intraventricular catheterization is implicated in hospital-acquired postoperative meningitis.^6^ This underscores the non-negligible role played by the lung microbiome in the pathological modifications of CNS disorders precipitated by exposure to environmental pathogens.

### 3.2 Neural pathways

Bidirectional communication along the brain-lung axis is primarily mediated by the autonomic nervous system, orchestrated by the synergistic interplay of the vagus nerve (parasympathetic) and the sympathetic nervous system. Vagal nerve endings are extensively distributed throughout the pulmonary parenchyma, facilitating bidirectional signal transduction via stimulation receptors and tension receptors located on sensory nerve fibers. These receptors transmit pulmonary signals to the CNS while concurrently receiving feedback signals from the CNS to modulate respiratory activity. Researchers have identified that GABAergic neurons within the central amygdala (CeA) exhibit pronounced activation during severe pneumonia, substantially contributing to inflammation and pulmonary injury. Inhibition of CeA GABAergic neurons mitigated the pulmonary cytokine storm and enhanced survival rates in murine models of lethal pneumonia. The CeA-rostral ventrolateral medulla-presympathetic neurons pathway establishes a conduit between the brain and lungs, potentiating norepinephrine (NE) release and amplifying inflammatory signaling.^7^

Investigations further reveal an association between the vagus nerve and allergic pathologies such as asthma and COPD, where it mediates bronchoconstriction and mucus hypersecretion. The vagus nerve may potentially serve as a conduit for pathogen dissemination: viruses infecting the lungs can access the brainstem respiratory center via the vagus nerve, while bacteria may exploit analogous routes or utilize outer membrane vesicles to breach the BBB.^3^ Additionally, experimental evidence indicates that activating pulmonary vagal sensory neurons can induce serotonin release from the dorsal raphe nucleus, ameliorating depressive behaviors, thereby suggesting the therapeutic potential of a "pulmonary vagus-brain axis".^8^ During asthma exacerbations, the paraventricular nucleus (PVN) and the dorsal vagal complex become activated. Oxytocin neurons within the PVN may participate in the pathogenesis of asthma attacks. Upon asthma onset, neuronal excitation within the PVN generates impulses that reflexively modulate peripheral airway hyperreactivity via the medullary respiratory center. Vagal ischemia induced by subarachnoid hemorrhage precipitates vagal axon degeneration, contributing to sympathetic-vagal imbalance and implicating this pathway in the pathogenesis of NPE.^9^

Altered functional activity within specific brain regions correlates with asthma. Utilizing resting-state functional MRI, alterations in activity have been detected in the cerebellum, frontal lobe, temporal lobe, occipital lobe, and insular cortex of asthma patients. During acute exacerbations, inflammation stimulates afferent fibers of the pulmonary vagus nerve, relaying signals to the nucleus tractus solitarius, which subsequently projects to multiple encephalic regions, influencing their functional dynamics. Concurrently, within the chronic inflammatory milieu of asthma, increased airway resistance stimulates respiratory reflexes, chemoreceptors, and pulmonary stretch receptors within the airways. The resultant neural impulses traverse the spinothalamic pathway to the insular cortex and anterior cingulate cortex, with respiratory modulation being integrated via functional connections involving the medial prefrontal cortex and the brainstem. Asthma attacks are frequently accompanied by varying degrees of hypoxia, which induces synaptic degeneration within the hippocampus, culminating in impaired learning, memory, and even cognitive dysfunction.^10^

**Fig. 2:**
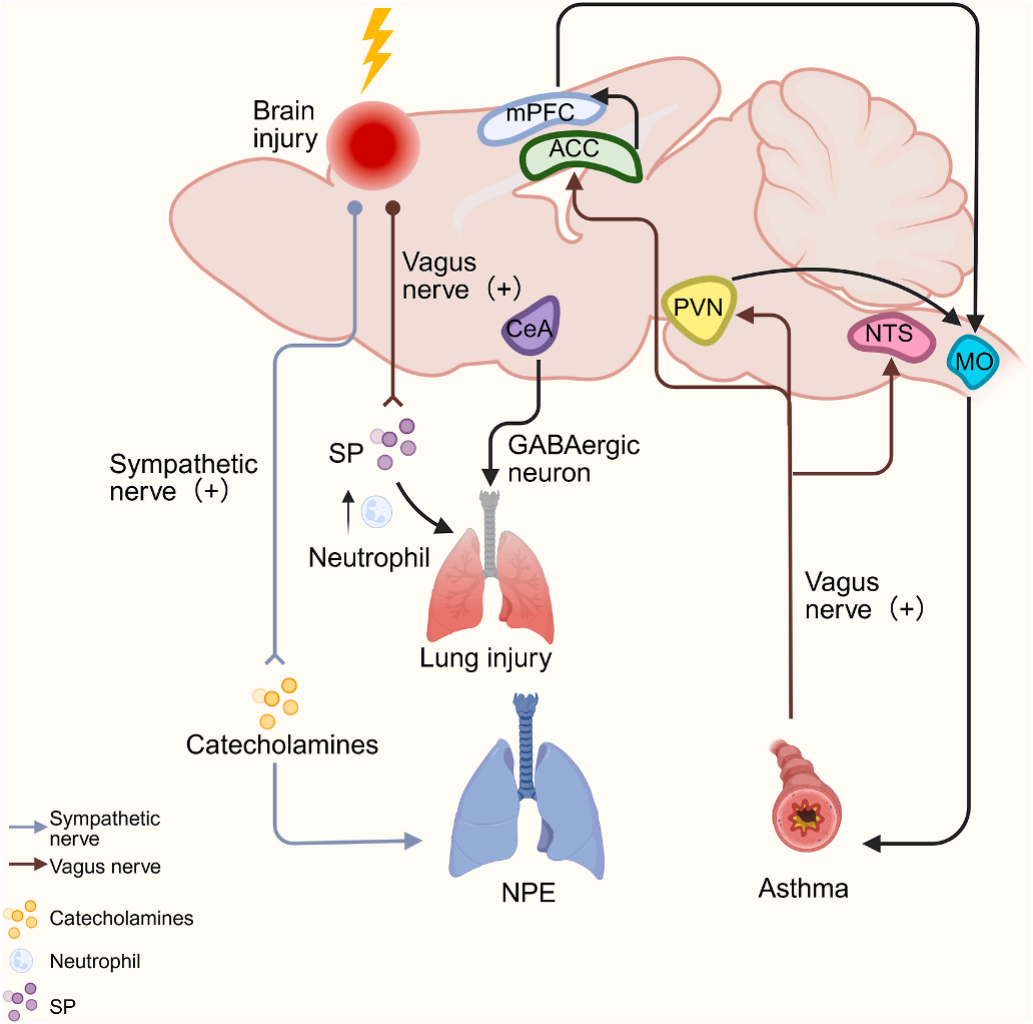
Schematic overview of neural pathways. Asthma activates multiple brain regions via the vagus nerve; influencing activity in the mPFC, ACC, PVN, MO, and NTS can subsequently modulate the medullary respiratory center. Brain injury triggers sympathetic activation, leading to catecholamine release and contributing to NPE. Additionally, brain-induced vagal activation releases SP, promoting neutrophil recruitment and ultimately causing lung injury. MO: medulla oblongata; mPFC: medial prefrontal cortex; ACC: anterior aingulate cortex; PVN: paraventricular nucleus of hypothalamus; CeA: central amygdaloid nucleus; NTS: solitary nucleus; NPE: neurogenic pulmonary edema; SP: substance P. Created with BioRender.com.

TBI triggers excessive sympathetic activation, instigating a catecholamine storm. Catecholamines, acting via α-adrenergic receptors, provoke pulmonary vasoconstriction and systemic vasoconstriction, thereby diverting blood volume into the pulmonary circulation and elevating pulmonary capillary hydrostatic pressure.^11^ Simultaneously, compromise of the BBB permits pro-inflammatory cytokines such as interleukin (IL)-6 and tumor necrosis factor (TNF)-α to enter the systemic circulation, activating pulmonary inflammation and promoting cellular apoptosis,^12^^,^^13^ ultimately culminating in NPE.^14^ Pretreatment with α-adrenergic antagonists can attenuate pulmonary injury, while β-blockers improve outcomes in TBI patients.^11^ Notably, the blast injury theory posits that a rapid surge in intracranial pressure damages the alveolar-capillary membrane via sympathetic storm, whereas the pulmonary venule hypersensitivity theory proposes that isolated pulmonary vasoconstriction can also precipitate NPE (Fig. 2).^13^

### 3.3 Metabolite pathways

The neuropeptide substance P (SP) is expressed in numerous (>30) brain regions. Microinjection of SP into the ventrolateral medulla elicits augmented respiratory amplitude concomitant with reduced frequency. Peripherally, SP contributes to airway inflammation by inducing bronchoconstriction and mucosal edema, thereby exhibiting a significant association with pathologies such as asthma. Following noxious stimuli (e.g., TBI), vagal afferent neurons secrete SP to mediate pulmonary vasodilation, enhanced vascular permeability, and neutrophil recruitment and migration. Conversely, blockade of SP receptors suppresses neutrophil influx, impairs bacterial clearance, and promotes the development of bacterial pneumonia.^15^ In asthmatic rat models, SP generated within the hypothalamic paraventricular nucleus not only reflexively modulates airway and pulmonary activity via medullary regions but also influences peripheral airway hyperreactivity through the regulation of hypothalamic-pituitary-adrenal (HPA) axis activity.

Extracellular vesicles (EVs), comprising heterogeneous membrane structures such as exosomes, microvesicles, and apoptotic bodies, participate in bidirectional communication between the brain and lungs under both physiological and pathological conditions.^16^ In murine models of TBI, brain-derived EVs, including brain-derived microvesicles, transport inflammatory proteins to the lungs. This process induces pyroptosis in pulmonary endothelial cells, compromises alveolar-capillary barrier integrity, and exacerbates vascular permeability, pulmonary edema, and infiltration of immune cells and cytokines.^17^ Conversely, inhibiting the uptake of brain-derived EVs in the lungs attenuates pulmonary inflammation.^18^ It is demonstrated that EVs containing inflammasome proteins induce acute lung injury in TBI mouse models. Serum-derived EVs from TBI patients carry inflammasome components, promoting pulmonary endothelial pyroptosis and disrupting barrier integrity, thereby aggravating vascular leakage and pulmonary edema.^19^

Hyperoxic conditions stimulate the release of lung-derived EVs containing inflammasome proteins, which subsequently enter the circulation of neonatal mice and rats, inducing neuroinflammatory injury.^20^ Studies have shown that preterm infants undergoing hyperoxia therapy exhibit significantly elevated levels of apoptosis-associated speck-like protein containing a caspase recruitment domain in alveolar macrophage-derived EVs. These apoptosis-associated speck-like protein containing a caspase recruitment domain-carrying EVs enter the bloodstream, cross the BBB, and reach key brain regions, such as the hippocampus. In mouse models, they trigger pulmonary inflammation, impaired alveolarization, and aberrant vascular development, while concurrently inducing neuroinflammation, cell death, and activation of pyrolytic and necroptotic pathways. This evidence underscores the role of EVs as interorgan messengers that transmit lung injury signals to the brain, serving as critical mediators in bronchopulmonary dysplasia and associated brain injury in preterm infants.^21^

It is reported that circulating blood exosomes containing caspase-1 cross the BBB, leading to neuroinflammation and caspase-1/gasdermin D activation in neonatal rats with ventilator-induced lung injury. This interaction may contribute to brain injury and neurodevelopmental deficits in infants subjected to injurious or prolonged mechanical ventilation.^19^ Both neurons and glial cells release EVs containing bioactive molecules that, upon secretion, regulate synaptic plasticity and activity, neurogenesis, myelination, and neuronal homeostasis. These EVs can traverse the BBB, modulating physiological recovery following disease or injury. CNS disorders, such as TBI, neurodegenerative diseases, and infections, are characterized by neuroinflammation. Under these conditions, altered EVs mediate pathway regulation, microglial responses, and secretion of pro-inflammatory cytokines, thereby either suppressing or resolving inflammatory reactions in recipient cells.^22^

Activation of the sympathetic-adrenomedullary axis, integral to the body's stress response, elevates plasma catecholamine levels, including epinephrine, NE, and dopamine. NE potentiates bacterial proliferation, enhances bacterial motility and toxin expression, thereby augmenting bacterial pathogenicity under stress and elevating the incidence of pulmonary infections.^15^

In the pathogenesis and progression of asthma, inflammatory cytokines stimulate the amygdala, activating the HPA axis. Circulating glucocorticoids subsequently bind to glucocorticoid receptors on airway epithelial and immune cell surfaces, modulating the expression of IL-4, IL-5, and IL-13, thereby effectuating central regulation of peripheral inflammation.^23^

### 3.4 Immune pathway

Microglia constitute the principal immune cells within the CNS. Upon inflammatory stimulation, microglia undergo activation into the M1 phenotype, releasing substantial quantities of pro-inflammatory mediators. These mediators inflict neuronal damage through multiple mechanisms, including the upregulation of indoleamine 2,3-dioxygenase, which diverts tryptophan metabolism away from serotonin synthesis towards the kynurenine pathway, ultimately culminating in neuronal demise. Concurrently, activated microglia engage in deleterious interactions with astrocytes, exacerbating neuroinflammation.^24^

Pulmonary dysbiosis exerts a profound influence on microglia, inducing morphological alterations characterized by reduced branching complexity and diminished responsiveness to inflammatory signals. This dysregulation results in diminished recruitment of immune cells to inflamed brain tissue. Investigators have discerned significant overlap between differentially expressed genes within total brain tissue and those specifically altered in microglia. Integrating data from gene ontology analysis and quantitative polymerase chain reaction techniques, these findings indicate that CNS tissue alterations induced by lung microbiome predominantly impact microglial function. This elucidates the mechanistic involvement of lung microbiome in brain autoimmune processes and furnishes novel evidence for the influence of peripheral organs on the CNS.^1^^,^^25^

Furthermore, the onset of oxidative stress in asthma modulates Th1/Th2 immune-inflammatory responses, activates microglia, and promotes the release of inflammatory cytokines within brain regions, such as the hippocampus via activation of the NF-κB signaling pathway. Consequently, this cascade reduces N-acetylaspartate levels in the hippocampi of asthmatic individuals, ultimately precipitating cognitive dysfunction.^10^

Following pulmonary infection or injury, immune cells release diverse pro-inflammatory mediators and regulatory factors. Notably, IL-6 induces microglial toll-like receptor 4/nuclear factor Kappa-light-chain-enhancer of activated B cells signaling during ventilator-induced lung injury, triggering apoptosis in frontal cortical cells—an effect reversible by IL-6 antibody administration. Conversely, lung-derived IL-4 induces phosphorylation of Claudin-5 in brain microvascular endothelial cells, augmenting BBB permeability through the impairment of tight junction integrity.^26^

Post-TBI, significant increases in Th1, CD4^+^, and CD8^+^ T cells occur, playing pivotal roles in pulmonary immunity.^27^ An elevated proportion of CD4^+^CD25^+^ regulatory T cells in peripheral blood potentially delays pulmonary tissue repair via suppression of Th1-type immune responses.^13^ Reduced memory B cell populations suggest their potential suppression or migration.^27^ Concurrently, microglial activation ensues, generating diverse pro-inflammatory molecules that translocate into the systemic circulation through a compromised BBB, ultimately inducing severe pulmonary injury.

## 4 Diseases associated with the brain-lung axis

**Fig. 3:**
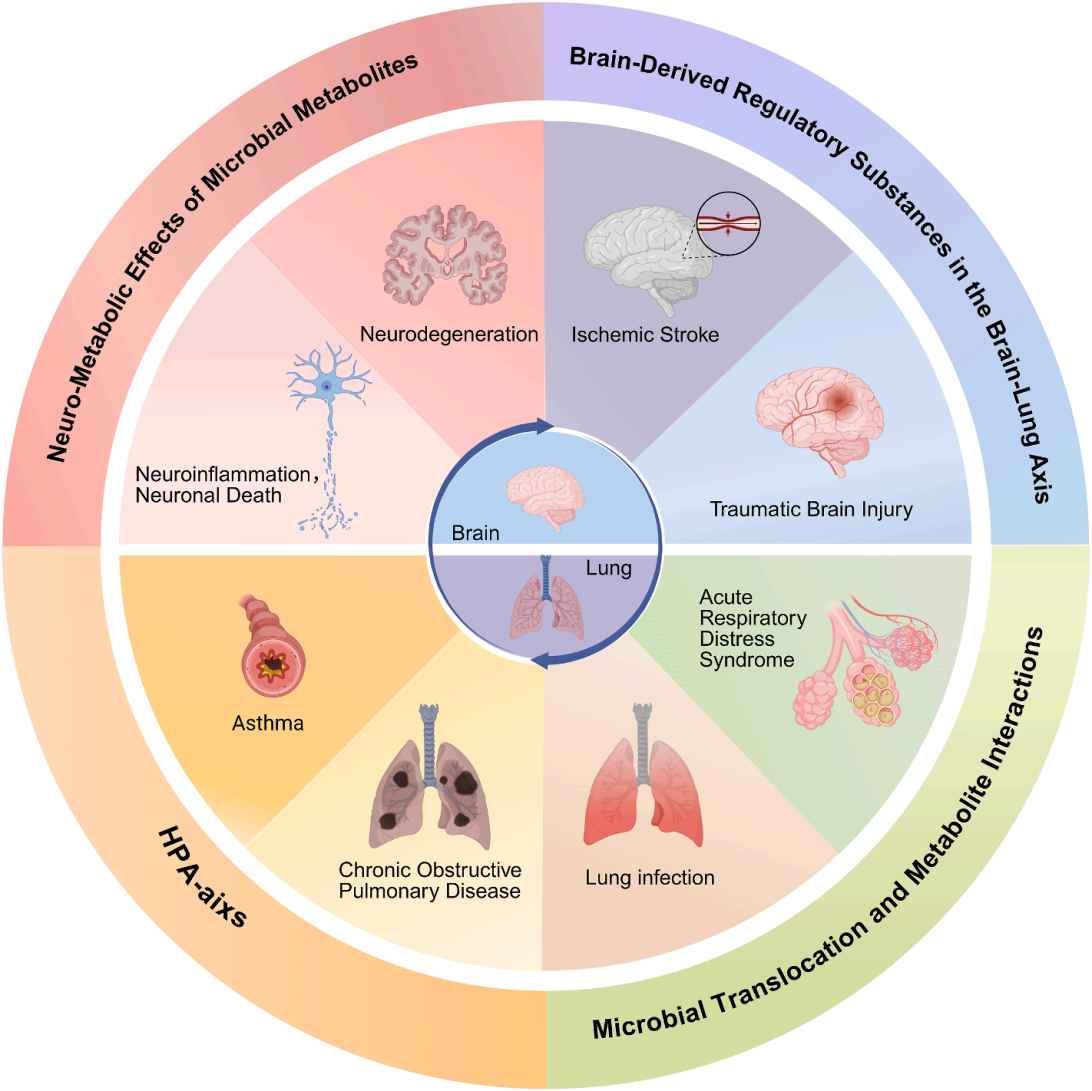
An outline map of brain–lung interactions via multiple pathways. This schematic illustrates the roles of the lung microbiome, along with neural, metabolic, and immune pathways, in contributing to brain pathologies—such as neurodegeneration, ischemic stroke, and traumatic brain injury—and lung diseases, including ARDS, asthma, COPD, and infection. These processes lead to neuroinflammation, neuronal death, and dysregulation of the HPA axis. ARDS: acute respiratory distress syndrome; COPD: chronic obstructive pulmonary disease; HPA: hypothalamic-pituitary-adrenal axis. Created with BioRender.com.

The brain and lungs form a bidirectional regulatory loop through pulmonary microbiota, neural, immune, and metabolic pathways. Brain injuries, such as stroke and TBI, can rapidly induce ARDS, NPE, and pulmonary infections via neural pathways, systemic inflammation, release of extracellular mitochondria, and immunosuppression, significantly increasing mortality. Conversely, severe pulmonary diseases, including ARDS and pneumonia, lead to cerebral ischemic injury, microbleeds, cognitive impairment, and delirium through systemic inflammatory storms, hypoxemia, and iatrogenic injury such as mechanical ventilation, thereby exacerbating neurological damage (Fig. 3).

### 4.1 TBI

TBI frequently precipitates diverse pulmonary complications, which may progress to ARDS in severe instances. ARDS manifests a bimodal distribution with an incidence of 20% – 30% and a mortality rate of 30% – 40%.^28^ Investigations employing controlled cortical impact murine models reveal that extracellular mitochondria (exMt) are released into bronchoalveolar lavage fluid post-TBI. These exMt exacerbate TBI-induced acute lung injury by promoting perivascular accumulation and activating pro-inflammatory macrophages that phagocytose exMt.^29^ Furthermore, post-injury serum factors directly compromise tight junctions in pulmonary endothelial cells, augmenting vascular permeability. TBI induces systemic oxidative stress, resulting in the excessive production of reactive oxygen species. As a highly perfused organ, the lung is particularly vulnerable to such oxidative damage, which exacerbates local inflammation and cellular apoptosis. Furthermore, TBI activates the HPA axis, leading to a substantial release of glucocorticoids. Concurrently, excitatory amino acids, such as glutamate enter the systemic circulation and exert direct detrimental effects on pulmonary cells, thereby contributing to lung injury. Additionally, brain injury triggers systemic immunosuppression, characterized by a reduction in T cells and natural killer cells, splenic atrophy, and lymphocyte apoptosis.^30^

TBI heightens susceptibility to pulmonary infections and clinical deterioration, exemplified by ventilator-associated pneumonia (VAP)—a prevalent iatrogenic pulmonary infection among mechanically ventilated critically ill patients. Severe TBI patients typically exhibit impaired consciousness and profound immunosuppression, collectively elevating VAP risk. Comparative analyses indicate increased VAP frequency in severe TBI patients relative to general pediatric intensive care unit populations.^31^

A pooled analysis revealed that the prevalence of ARDS among TBI patients reaches 19%, indicating that approximately 1 in every 5 patients develops ARDS at a median time of 3 days post-injury. This highlights the rapid impact of brain injury on remote pulmonary organs. In terms of clinical outcomes, TBI patients complicated with ARDS exhibit significantly lower hospital discharge survival rates and a reduced proportion of favorable neurological recovery compared to those without ARDS, clearly demonstrating the adverse effect of ARDS on TBI prognosis.^32^

Systemic inflammation and hypoxia consequent to ARDS can precipitate secondary brain injury. MRI of patients hospitalized with ARDS reveals that 44% exhibit structural brain abnormalities, most commonly ischemic injuries, such as bilateral globus pallidus infarctions, multifocal embolic infarctions, and diffuse hypoxic-ischemic injuries, and brain hemorrhages, particularly brain microbleeds and intraparenchymal hemorrhages.^33^^,^^34^ Pulmonary injury instigates a downstream cytokine storm, resulting in neutropenia and activation of pulmonary dendritic cells and macrophages. This cascade exacerbates localized inflammation, impairs end-organ function—including brain dysfunction—and may trigger autoimmune encephalitis. Consequently, neuronal activity and brain perfusion are compromised, culminating in hypoxic-ischemic brain injury.^35^^,^^36^

### 4.2 Stroke

Post-stroke pulmonary complications, collectively termed stroke-associated lung injury, may arise from direct damage or functional impairment of the vagus nerve. Such vagal dysfunction compromises the cholinergic anti-inflammatory pathway, thereby promoting systemic inflammation. Concurrently, the ischemic brain tissue releases substantial amounts of pro-inflammatory cytokines into the systemic circulation, which contribute to a generalized inflammatory state and subsequently target the lungs.^37^ Stroke patients frequently exhibit impaired respiratory muscle function and reduced performance on pulmonary function tests. Beyond these deficits, an intense inflammatory cascade may erupt intracranially, while peripheral immunosuppression increases susceptibility to respiratory infections.^38^ Among these complications, stroke-associated pneumonia represents the most prevalent and frequently fatal outcome. Clinical evidence confirms that stroke-induced immunosuppression syndrome is an independent risk factor for stroke-associated pneumonia. Approximately 1 in 10 stroke patients develops pneumonia during the acute phase. In cases of severe stroke, medullary ischemia triggers a sympathetic storm, leading to pulmonary vasoconstriction and elevated capillary hydrostatic pressure, which can rapidly progress to ARDS within 48 h.^39^ More than 50% of major stroke patients develop ARDS within 72 h of admission to the neurological intensive care unit, through mechanisms involving inflammatory cytokine spillover, coagulopathy, and autonomic dysregulation.^9^ Additionally, NPE is another common pulmonary complication following stroke.^40^ Many severe stroke patients require mechanical ventilation (MV) for airway protection. However, MV itself can induce lung injury and subsequently trigger neuronal activation across multiple brain regions through mechanisms involving neutrophil infiltration and release of inflammatory mediators. These processes alter cerebral perfusion and activate the autonomic nervous system. Studies indicate that MV exerts detrimental effects on the hippocampus via the transient receptor potential vanilloid 4-adenosine triphosphate-purinergic receptor P2X4 signaling pathway in lung tissue, ultimately contributing to ventilator-associated cognitive impairment.^37^

Stroke often ensues after ARDS. In ARDS patients, diffuse cerebral microbleeds predominantly affect the cerebellum, brainstem, and juxtacortical white matter. These erebral microbleeds possess the potential to evolve into hemorrhagic stroke. During a hemorrhagic stroke, substantial blood influx into the ventricular cavities induces a precipitous rise in intracranial pressure (ICP), subsequently compressing the brainstem and medulla, thereby inducing ischemia. As the principal trigger zone for NPE, stimulation of the medulla prompts the sympathetic nervous system to release massive quantities of catecholamines. This exerts profound effects on the pulmonary system, augmenting pulmonary vascular resistance, elevating pulmonary arterial pressure and capillary hydrostatic pressure, and ultimately instigating pulmonary congestion and edema. Notably, SARS-CoV-2 pulmonary infection downregulates angiotensin-converting enzyme 2 receptor expression, thereby augmenting the risk of hemorrhagic stroke.^41^ Watershed infarctions during septic shock and gas embolism secondary to mechanical ventilation barotrauma may precipitate ischemic stroke. Prolonged hypoxemia resulting from ARDS, coupled with hypotension induced by concomitant sepsis and shock, disrupts oxygen and glucose delivery to brain tissues. This culminates in hypoxic-ischemic brain injury, particularly within structures such as the hippocampus.^9^

### 4.3 Other disorders

Respiratory compromise is reported in up to 40% of patients with Parkinson's disease.^42^ Individuals with Parkinson's disease may exhibit impaired respiratory function even in early stages,^43^ manifesting as respiratory muscle weakness, elevated hospitalization rates for pneumonia (excluding aspiration pneumonia) and respiratory infections, alongside increased respiratory morbidity and mortality.^44^

Pulmonary infections and inflammation can activate peripheral immune cells, leading to the release of inflammatory mediators such as ILs and TNFs. These mediators can cross the BBB, disrupt neural cells and neurotransmitters in the brain, and thereby contribute to the pathogenesis of AD.^5^ Smoking-induced pulmonary irritation and inflammation may participate in the initiation and progression of MS via the brain-lung axis.^45^

Beyond their profound impact on the respiratory system, coronaviruses—particularly SARS-CoV-2—exhibit neuroinvasive potential. After invading the CNS, the virus alters metabolism in infected and adjacent neuronal populations, resulting in focal cerebral ischemia and cell death. SARS-CoV-2-infected patients frequently develop neurobehavioral symptoms, including anxiety, depression, and cognitive impairment.^46^^,^^47^ Notably, the virus has also been shown to induce acute anosmia and Alzheimer's-like pathology in Syrian golden hamsters.^48^

Patients with bipolar disorder or major depressive disorder exhibit an elevated risk of developing pulmonary conditions, such as COPD and asthma.^49^^,^^50^ Most studies indicate that the prevalence of mental health disorders (e.g., anxiety and depression) and cognitive impairment in COPD patients is 2 to 3 times higher than in healthy subjects.^51^ Compared to other chronic diseases, the incidence of anxiety and depression is even greater among COPD patients, and the comorbidity of COPD with depression/anxiety is associated with increased mortality risk. Depressive and anxious symptoms may diminish patients' quality of life and treatment adherence,^3^ while also prolonging hospitalization during COPD exacerbations.^52^

ARDS survivors frequently experience cognitive impairment and functional disability, which compromise quality of life and increase susceptibility to intensive care unit delirium.^53^ Substantial evidence indicates that asthma patients present not only with respiratory symptoms but also with prevalent neuropsychiatric manifestations, including cognitive decline, anxiety, and sleep disturbances. Conversely, psychological stress and negative emotions can activate cerebral regions, such as the anterior cingulate cortex, insula, and limbic system, triggering the release of neuroactive molecules, including SP, histamine, and neuropeptide Y. These molecules propagate via specialized pathways to the airways, where they interact with peripheral nerves and immune cells to exacerbate asthmatic symptoms—thereby establishing a self-perpetuating pathological cycle.^54^^,^^55^ Concurrent reductions in hippocampal N-acetylaspartate metabolites^56^ further substantiate the presence of cognitive dysfunction and impaired emotional regulation.^54^^,^^57^

## 5 Treatment strategies

### 5.1 TBI

The management of elevated intracranial pressure follows a stepwise and integrated therapeutic protocol. Foundational measures include elevating the head of the bed to 30° – 45° to facilitate venous drainage, along with adequate sedation and analgesia to reduce cerebral metabolic demand, control agitation, and stabilize intracranial pressure. For persistent elevation, ventricular cerebrospinal fluid drainage or osmotic therapy using hypertonic agents such as mannitol or hypertonic saline may be considered, with close monitoring of plasma osmolality and electrolyte balance. Hyperventilation may serve as a temporary rescue measure by reducing arterial carbon dioxide partial pressure to induce cerebral vasoconstriction and decrease intracranial blood volume, though excessive use should be avoided to prevent cerebral ischemia. In cases of refractory intracranial hypertension resistant to pharmacological intervention, mild therapeutic hypothermia may be applied. Should all aforementioned measures prove ineffective and the patient's anticipated quality of life remains acceptable, decompressive craniectomy is recommended as a definitive surgical intervention; however, it is generally not suitable for patients in late-stage cerebral herniation.^58^

The core objectives of neurocritical care involve maintaining intracranial pressure below 20 – 25 mmHg and cerebral perfusion pressure between 60 mmHg and 70 mmHg through multimodal monitoring, while managing ventilation, sedation, osmotic therapy, and preventing complications, such as seizures and deep vein thrombosis. Long-term management entails neuroendocrine screening, cognitive and behavioral rehabilitation, and cranioplasty. Additionally, pharmacotherapy for post-traumatic epilepsy, the clinical utility of brain tissue oxygen monitoring, and the application of artificial intelligence in personalized treatment represent important directions for future research.^59^

Advanced cardiovascular monitoring and support are routinely implemented in patients with TBI. Cerebral oxygenation is monitored via PbtO_2_ measurement while avoiding hypocapnia secondary to elevated ICP to minimize ischemic risk. Close monitoring of elevated ICP is essential, often combined with invasive neuromonitoring and cerebral perfusion pressure management.^60^ Therapeutic hypothermia may be utilized to reduce ICP, attenuate inflammatory responses, and lower cerebral metabolic rate, thereby conferring neuroprotective benefits and mitigating TBI consequences.^61^ When necessary, decompressive craniectomy effectively reduces ICP, as does the administration of barbiturates.^62^

Hyperbaric oxygen therapy has been demonstrated to ameliorate cognitive deficits in severe TBI, elevate levels of BDNF nerve growth factor, and vascular endothelial growth factor, promote vascular repair, and improve patient outcomes.^63^ Temporary transvenous diaphragmatic neurostimulation reduces hippocampal apoptosis and inflammation following 50 h of mechanical ventilation.^64^ Studies indicate that optimizing high positive end-expiratory pressure, tidal volume, and oxygenation parameters is critical for maintaining cerebral perfusion pressure and neurological function.^36^^,^^65^

### 5.2 Stroke

In terms of acute phase management, the guidelines have significantly updated reperfusion strategies. Intravenous thrombolysis with alteplase or tenecteplase is strongly recommended for ischemic stroke patients within 4.5 h of symptom onset. Thrombolysis should also be considered for patients presenting between 4.5 and 9 h or with wake-up stroke, provided neuroimaging (CT perfusion/MRI) demonstrates salvageable brain tissue. The indications for mechanical thrombectomy have been substantially expanded, now including selected patients with anterior circulation large vessel occlusion within 6 – 24 h from onset (based on Alberta stroke program early CT score and perfusion mismatch), as well as those with posterior circulation large vessel occlusion within 12 h.

For transient ischemic attack and minor stroke, short-term dual antiplatelet therapy (aspirin combined with clopidogrel or ticagrelor) initiated within 24 h is strongly recommended to significantly reduce early recurrence risk. In spontaneous intracerebral hemorrhage management, urgent intensive blood pressure lowering is recommended for patients with systolic blood pressure between 150 mmHg and 220 mmHg within 6 h of onset, targeting a reduction to 130 – 139 mmHg within 1 h and maintaining this for at least 7 days. Early non-invasive vascular imaging is advised for specific patient groups to screen for underlying macrovascular causes.^66^

Following revascularization, to prevent hyperperfusion syndrome and hemorrhagic transformation, strict systolic blood pressure control at 140 – 180 mmHg should be implemented immediately, avoiding intensive reduction below 120 mmHg. For patients on anticoagulants (e.g., warfarin, dabigatran, factor Xa inhibitors), emergency administration of specific reversal agents (such as vitamin K, idarucizumab, andexanet alfa) is required. Medications that increase bleeding risk should be avoided. Management of severe hemorrhagic transformation primarily involves surgical intervention and symptomatic support, focusing on intracranial pressure control and vital sign stabilization. For procedure-related complications, such as vascular rupture during endovascular therapy, immediate balloon occlusion or coil embolization can be employed for hemostasis. Decompressive craniectomy becomes necessary when severe postoperative intracranial hemorrhage with mass effect occurs.^67^ Supportive care and secondary prevention are implemented concurrently, supplemented by neuroprotective agents including butylphthalide and ApTOLL.^68^

Post-stroke activation of the pulmonary sympathetic nervous system leads to catecholamine release, which reduces TNF-α levels and elevates IL-10 levels via stimulation of β-adrenergic receptors on immune cells. Blockade of the sympathetic nervous system using β-adrenergic receptor antagonists can mitigate infectious complications, such as pneumonia, and reduce mortality rates.^15^ Accumulating evidence indicates that hyperbaric oxygen therapy promotes neuroplasticity, improves cognitive function and quality of life in patients with chronic neurocognitive impairments resulting from TBI, stroke, or hypoxic brain injury, and attenuates neuroinflammation,^69^ while also exhibiting neuroprotective, antioxidant, and anti-apoptotic properties.^70^, ^71^, ^72^

### 5.3 Other disorders

The conventional management of AD typically involves pharmacological interventions, such as anti-Aβ monoclonal antibodies (e.g., Aducanumab, Lecanemab),^73^^,^^74^ N-Methyl-D-Aspartate receptor antagonists (e.g., Memantine), and acetylcholinesterase inhibitors (Donepezil, Galantamine, and Rivastigmine).^75^ Treatment for Parkinson's disease commonly employs Levodopa, dopamine receptor agonists, or monoamine oxidase-B inhibitors.^76^ Deep brain stimulation is widely utilized in Parkinson's disease management and has also been applied in treating pain syndromes, depression, AD, anorexia nervosa, and schizophrenia.^77^

Respiratory drug delivery refers to nasal and pulmonary administration routes that bypass hepatic first-pass elimination and facilitate penetration across the BBB into the CNS. This approach enables direct delivery to brain tissue or cerebrospinal fluid via olfactory neurons. The lungs' extensive surface area, thin alveolar epithelium, and abundant blood supply promote rapid and efficient drug absorption. Experimental studies demonstrate that inhaled sea buckthorn seed oil improves memory and cognitive function in AD mouse models,^78^ while inhaled Levodopa has been investigated for Parkinson's disease treatment.^79^ Additional reports indicate the promising efficacy of respiratory-delivered CNS-targeted drugs in managing anxiety and migraine.^80^

For psychiatric disorders, standard pharmacological interventions include selective serotonin reuptake inhibitors (e.g., fluoxetine, escitalopram), serotonin and norepinephrine reuptake inhibitors (e.g., venlafaxine), and benzodiazepines (e.g., alprazolam).^81^ Physical modalities such as transcranial magnetic stimulation^82^ and electroconvulsive therapy^83^ also represent effective interventions. Currently, cognitive behavioral therapy remains the most widely available and empirically supported psychotherapy for major depressive disorder,^84^ while mindfulness-based stress reduction demonstrates favorable safety and tolerability with efficacy comparable to first-line pharmacotherapy like escitalopram for anxiety disorders.^85^

Research indicates that mesenchymal stromal cells exert antidepressant and anxiolytic effects through BDNF-TrkB axis-mediated activation of 5-HT neurons in the dorsal raphe nucleus via pulmonary afferent nerves. Inhaled administration of the TrkB agonist 7,8-DHF ameliorates depressive and anxiety-like behaviors, suggesting a potential therapeutic pathway for depression.^86^^,^^87^

A bidirectional therapeutic relationship exists between brain and pulmonary rehabilitation. Respiratory training enhances hippocampal and prefrontal function through vagal nerve activation, improving cognition and emotion, while neurorehabilitative training (e.g., attentional control) improves respiratory control, establishing a positive feedback cycle. Integrated rehabilitation protocols combining breathing exercises with cognitive training may overcome single-organ therapy limitations and enhance quality of life in chronic respiratory diseases.^88^ Exercise therapy not only improves cardiopulmonary function but also ameliorates immunometabolic depression.^89^ For patients with COPD comorbid with depression and anxiety, pulmonary rehabilitation—a specialized COPD intervention—confers beneficial effects on anxiety and depression alongside conventional psychological and pharmacological treatments.^52^

## 6 Conclusions and prospects

The brain-lung axis serves as a pivotal hub of intricate interactions between the CNS and the lungs, illuminating the bidirectional regulatory mechanisms that underlie both physiological homeostasis and pathological progression. Microbial and neural pathways constitute the core mechanisms mediating cross-talk between the brain and lungs. This interplay is not merely an extension of local pathology, but rather a critical driver of systemic multisystem dysfunction.

Although significant advances have been made in brain-lung axis research, several avenues warrant further exploration. These include employing single-cell sequencing, spatial transcriptomics, and neural tracing technologies to elucidate dynamic interactions at the molecular and cellular levels, thereby deepening the mechanistic understanding of lung microbiome metabolites. There is also a need to develop more combined brain-lung therapeutic strategies, transcending disciplinary boundaries by integrating neuroscience, respiratory medicine, and microbiology to facilitate the translation of mechanistic insights into clinical interventions. Targeted modulation of this axis holds promise for pioneering novel therapeutic paradigms for both respiratory and neurological disorders, ultimately improving patient quality of life and reducing the societal burden of disease.

## CRediT authorship contribution statement

**Wenpei Yang:** Writing – original draft. **Xiaoyu Shi:** Visualization. **Lizheng Xie:** Writing – review & editing. **Hao Zhang:** Methodology. **Lina Shen:** Data curation. **Li Pan:** Methodology. **Shi Feng:** Formal analysis. **Xiang Mao:** Writing – review & editing, Supervision, Funding acquisition, Data curation, Conceptualization. **Xiao Wu:** Conceptualization.

## Ethical statement

Not applicable.

## Funding

The funding agencies, including grants from National Natural Science Foundation of China (No.82202438), Clinical and Translational Research Project of Anhui Province (No.202427b10020130), Beijing Postdoctoral Foundation (No.2020-ZZ-008), Returned overseas students from Anhui Province in 2020 Innovation and Entrepreneurship Support Plan Project (No.2020LCX016), Funding of The Anhui Universal Natural Science Foundation (No.KJ2020A0172), Experimental and Clinical Cooperative Research Advanced Program of Anhui Medical University (No.2022xkjT032), Cultivate Funding of the National Natural Science Foundation of China (No.2016KJ12). We are very thankful to all the study participants and acknowledge the core management group for organizing the databases.

## Declaration of competing interests

All the authors declare that they have no competing financial interests.
